# Electrolyte disorders assessment in solid tumor patients treated with anti-EGFR monoclonal antibodies: a pooled analysis of 25 randomized clinical trials

**DOI:** 10.1007/s13277-014-2983-9

**Published:** 2014-12-28

**Authors:** Qiaoli Wang, Yuexiao Qi, Di Zhang, Caifeng Gong, Anqi Yao, Yi Xiao, Jie Yang, Fuxiang Zhou, Yunfeng Zhou

**Affiliations:** 1 Department of Radiation Oncology and Medical Oncology, Zhongnan Hospital of Wuhan University, Hubei Key Laboratory of Tumor Biological Behaviors and Hubei Cancer Clinical Study Center, No. 169 Donghu Road, Wuhan, Hubei 430071 People’s Republic of China; 2 Department of Oncology, The First Affiliated Hospital of Soochow University, No. 188 Shizi Street, Suzhou, Jiangsu 215006 People’s Republic of China

**Keywords:** Electrolyte disorders, Cetuximab, Panitumumab, Meta-analysis

## Abstract

The role of anti-epithelial growth factor receptor monoclonal antibodies (anti-EGFR MoAbs) in treatment-related electrolyte disorders is still controversial. Therefore, we conducted a meta-analysis of published randomized controlled trials (RCTs) to evaluate the incidences and overall risks of all-grade and grade 3/4 electrolyte disorder events. We searched relevant clinical trials from PubMed, EMBASE, and Web of Knowledge databases, meeting proceedings of American Society of Clinical Oncology and the European Society of Medical Oncology, as well as ClinicalTrials.gov. Eligible studies included phases II, III, and IV RCTs. Statistical analysis was performed to calculate the summary incidence, relative risk (RR), and 95 % confidence intervals (CIs) using fixed effects or random effects models based on the heterogeneity of included studies. A total of 16,411 patients from 25 RCTs were included in this meta-analysis. The all-grade incidence of hypomagnesemia related to anti-EGFR MoAbs was 34.0 % (95 % CI 28.0–40.5 %), and that for hypokalemia and hypocalcemia were 14.5 % (95 % CI 8.2–24.4 %) and 16.8 % (95 % CI 14.2–19.7 %), respectively. Compared with chemotherapy alone in colorectal cancer, addition of cetuximab increased the risk of grade 3/4 hypomagnesemia and grade 3/4 hypokalemia with RRs of 7.14 (95 % CI 3.13–16.27, *p* < 0.001) and 2.19 (95 % CI 1.14–4.23, *p* = 0.019). Additionally, colorectal cancer patients in panitumumab cases were more vulnerable to grade 3/4 hypomagnesemia and hypokalemia (RR 18.29, 95 % CI 7.29–48.41, *p* < 0.001, and RR 3.3, 95 % CI 1.32–8.25, *p* = 0.011). Treatment with anti-EGFR MoAbs is associated with significantly higher risks of electrolyte disorders such as hypomagnesemia, hypomagnesemia, and hypocalcemia, especially in colorectal cancer. Rigorous monitoring and early treatment of electrolyte disorders are proposed.

**Electronic supplementary material:**

The online version of this article (doi:10.1007/s13277-014-2983-9) contains supplementary material, which is available to authorized users.

## Introduction

Epithelial growth factor receptor (EGFR), which is also known as erbB1 or HER1, is the first growth factor receptor to be proposed as a target for anti-cancer therapy [1]. EGFR is a 170-kDa transmembrane protein with an intracellular tyrosine-kinase, which can be overexpressed by a range of different tumors such as colorectal cancer, head and neck cancer, lung cancer, pancreas cancer, and breast cancer [2]. It is crucial in modulating cellular signaling pathways including proliferation, inhibition of apoptosis, angiogenesis, invasion, and metastasis, making it a promising target for anti-cancer agent [3]. At present, anti-EGFR agents mainly include two types: tyrosine-kinase inhibitors (TKI) and monoclonal antibodies (MoAbs) [4]. The MoAbs approved by the US Food and Drug Administration (FDA) include cetuximab (Erbitux™), a chimeric immunoglobulin G1 antibody, in February 2004 and panitumumab (Vectibix™), a fully-human immunoglobulin G2 antibody, in September 2006 [5, 6]. These agents are still being evaluated in treatment of various advanced malignant diseases such as colorectal cancer, non-small-cell lung cancer (NSCLC), head and neck cancer, and so on. Thus, more applications of MoAbs are expected in the near future.

With respect to side effects, the most specific and frequently toxic effect of anti-EGFR MoAbs is acneiform eruption, skin rash, and other cutaneous events. They have been regarded as typical class adverse events related to MoAbs [7]. However, electrolyte disorders are also common adverse events during anticancer therapy but are often overlooked. If decreased electrolytes have not been managed timely, fetal events like cardiac arrhythmia, coronary artery vasospasm, and sudden cardiac death might take place. What is more, on account of lacking monitoring system, it would be more dangerous in outpatients.

Since the indications for anti-EGFR MoAbs are increasing, it is prerequisite to recognize the patterns of toxic effects such as incidence and relative risk (RR) of electrolyte disorder events and to understand the mechanism of the drug, so that early and essential intervention can be done. To our knowledge, on account of the limited number of patients in trials, there is no clinical trial with a great capacity to explore electrolyte disorders associated with MoAbs agents in detail. Thus, in order to better understand the overall risk of electrolyte disorders, we conducted a meta-analysis of published randomized controlled trials (RCTs) to investigate the incidence and RR of all-grade and grade 3/4 electrolyte disorders during the treatment of anti-EGFR MoAbs (cetuximab or panitumumab).

## Methods

### Search strategy 

This study was performed in accordance with Preferred Reporting Items for Systematic Reviews and Meta-Analyses (PRISMA) statement [8]. The first two authors independently conducted a comprehensive literature search of PubMed (January 1, 1966 to June 30, 2014) using the following keywords: “panitumumab,” “Vectibix,” “ABX-EGF” or “cetuximab,” “Erbitux,” “C-255,” limited by “clinical trial.” Then, we manually searched bibliographies of included trials with keywords of “randomized controlled trial” and “adverse events.” The same keywords were used to search abstracts and virtual meeting presentations from the American Society of Clinical Oncology (ASCO) and the European Society of Medical Oncology (ESMO) conferences. Information on ongoing registered clinical trials from the National Institutes of Health Web site (http://www.clinicaltrials.gov) was also referenced. The search strategy was also carried out to search the database EMBASE and Web of Knowledge to make sure that no relevant trials were neglected. Only the most recent, complete and full manuscripts from clinical trials were included.

### Eligibility criteria

The principal objective of this study was to determine the incidence and the overall risk of electrolyte disorders associated with anti-EGFR MoAbs. Thus, trials that matched the following criteria were included: (1) participating patients with all solid tumors at baseline; (2) randomized controlled phases II, III, and IV clinical trials; (3) patients were assigned to cetuximab or panitumumab therapy and controls; and (4) data available for the events of electrolyte disorders and sample size for analysis. We excluded studies if they met the following criteria: (1) phase I trials or single arm phase II trials that lack of controls; (2) any meta-analysis, comment, review, and case report; (3) retrospective trials; and (4) trials lack of suitable data of electrolyte disorders.

### Data extraction and study quality assessment

Two authors independently reviewed the full studies and the following information were included into an electronic database: name of first author, year of publication, trial phase, number of patients enrolled and analyzed, patients status, follow-up duration, underlying malignancy, treatment methods, National Cancer Institute’s Common Terminology Criteria for Adverse Events (CTCAE) criteria version, and adverse outcomes of events interest (hypomagnesemia, hypokalemia, hypocalcemia, and hyponatremia). We included the exact number of patients who occurred adverse events interest of all-grade and grade 3/4 and number of total patients enrolled in the clinical trials. The study quality was assessed by the same two reviewers independently according to the Jadad score which included randomization, blinding, and withdrawals, ranging from 0 to 5 points [9]. Any discrepancies were resolved by joint review of the manuscript to reach consensus.

### Statistical analysis

We used Comprehensive Meta Analysis program version 2 (Biostat, Engle-wood, NJ, USA) to pool data. The incidence, RR, and their 95 % confidence intervals (CIs) of adverse events were calculated for each study, and the results were compared through both random effects model (Der-Simonian and Laird’s method [10]) and fixed effects model (Mantel-Haenszel method). Statistical heterogeneity among studies was assessed by Cochrane’s *Q* statistic and *I* ^2^ statistic [11]. The *I* ^2^ value provides an estimate of amount of variance across studies derived from heterogeneity rather than chance. If *p* value of Cochrane’s *Q* statistic <0.1, the assumption of homogeneity was deemed invalid and a random effects model was reported; otherwise, results from the fixed effect model were reported. RR >1 reflects a higher overall risk of adverse events. All *p* values were two-tailed and were considered statistically significant if *p* < 0.05.

Subgroup analysis was performed by tumor type and MoAbs agent category. Sensitivity analysis was based on the weight or quality of the studies to assess the robustness of primary results. Publication bias was evaluated using Begg’s and Egger’s tests [12, 13].

## Results

### Literature search results

**Fig. 1:**
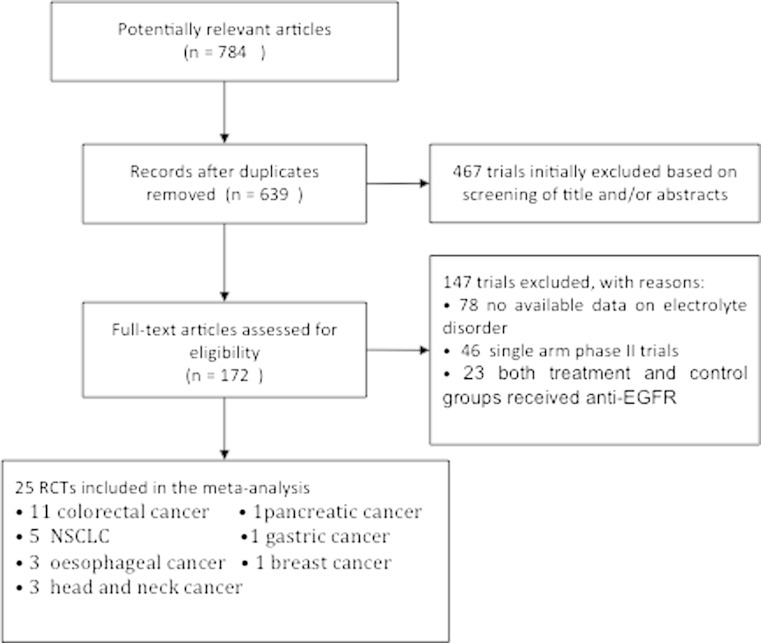
The literature search process

Seven hundred eighty-four potentially relevant clinical trials with anti-EGFR MoAbs were identified with the search strategy, of which 145 were initially excluded as duplicates (Fig. 1). After a review of the titles and abstracts of the remaining 639 publications, 172 trials were judged as promising articles. These articles were selected and evaluated in greater detail by reviewing the full articles. And, finally, 25 RCTs were considered as highly relevant trials for the meta-analysis.

### Study characteristics

Twenty-five RCTs reporting 23,094 patients were identified, among which, 16,411 were actually exposed to the original study. There were 3011 total electrolyte disorder events among these patients (anti-EGFR MoAbs, *n* = 2161; controls, *n* = 850). Table 1 shows the baseline characteristics of each trial. Underlying malignancies included colorectal cancer (11 studies) [14–24], NSCLC (five studies) [25–29], head and neck cancer (three studies) [30–32], oesophageal cancer (three studies) [33–35], pancreatic cancer (one study) [36], gastric cancer (one study) [37], and breast cancer (one study) [38]. National Cancer Institute’s Common Terminology Criteria for Adverse Events (CTCAE) criteria, version 2, 3, or 4 were used to evaluate the adverse events in these studies. The differences between the three versions were presented in Supporting Information Table 1 (Online Resource). These trials include 4 phase II [18, 26, 27, 38], 18 phase III [14–17, 19, 21–25, 28–32, 34, 36, 37], and 2 phase II/III studies [33, 35], and one study did not report the exact phase [20]. Thirteen studies mentioned follow-up duration, and 21 reported hypomagnesemia events, 16 studies for hypokalemia events, four for hypocalcemia events, and three for hyponatremia events as shown in Table 1. In all trials, patients were randomly assigned to with or without MoAbs-treated groups, according to Eastern Cooperative Oncology Group performance status or other criteria. Patients enrolled in the MoAbs group received cetuximab 400 mg/m^2^ at first dose and 250 mg/m^2^ per week (or 500 mg/m^2^ every 2 weeks) or panitumumab 6 mg/kg (or 9 mg/kg according to the tumor types) on day 1 of each cycle. The overall methodological study quality was generally good and fair with a Jadad mean score of 3.04, ranging from 2 to 5.

**Table 1:** Baseline characteristics of the 25 trials included in the meta-analysis

Author/year	Trial phase	Enrolled^a^	Analyzed^a^	Patients status	Follow-up (months)	Underlying malignancy	Treatment arms	Cet or Pan dose^b^	CTCAE version	Study quality^c^	AE interested
Jonker et al. (2007)	III	572	457	ECOG: 0–2	14.6	Colorectal cancer	BSC+Cet;	400,250	2	3	Mg
							BSC				
Tol et al. (2007)	III	755	389	WHO: 0–1	6.8	Colorectal cancer	Cap+Oxa+Bev+Cet;	400,250	3	3	Mg
							Cap+Oxa+Bev				
Maughan et al. (2011)	III	2445	1630	WHO: 0–2	NA	Colorectal cancer	Oxa+FU/Cap+Cet;	400,250	3	3	Mg
							Oxa+FU/Cap				
Alberts et al. (2012)	III	3661	2534	ECOG: 0–2	28	Colon cancer	Oxa+Leu+FU+Cet;	400,250	3	3	Mg
							Oxa+Leu+Flu				
Siena et al. (2013)	II	58	42	ECOG: 0–1	NA	Colorectal cancer	Len+Cet;	400,250	4	2	Mg; K
							Len				
Sobrero et al. (2008)	III	1587	1267	ECOG: 0–2	NA	Colorectal cancer	Iri+Cet;	400,250	2	3	Mg; K; Ca
							Iri				
Primrose et al.(2014)	NA	621	271	WHO: 0–2	20.7	Colorectal cancer	Oxa/Iri+FU/Cap+Cet;	500/400, 250	NA	3	Mg; K
							Oxa/Iri+FU/Cap				
Douillard et al. (2010)	III	1378	1084	ECOG: 0–2	NA	Colorectal cancer	FU+Leu+Oxa+Pan;	6	3	3	Mg; K
							FU+Leu+Oxa				
Peeters et al. (2010)	III	1345	1079	ECOG: 0–2	NA	Colorectal cancer	FU+Leu+Iri+Pan;	6	3	3	Mg; K
							FU+Leu+Iri				
Hecht et al. (2009)	IIIB	1240	804	ECOG: 0–1	7.5	Colorectal cancer	FU+Leu+Oxa/Iri+Bev+Pan;	6	3	3	Mg
					6.2		FU+Leu+Oxa/Iri+Bev				
Van Cutsem et al. (2007)	III	1040	463	ECOG: 0–2	NA	Colorectal cancer	BSC+Pan;	6	3	3	Mg
							BSC				
Kim et al.(2013)	III	939	605	Karnofsky: 60–100	NA	NSCLC	Pem+Cet;	400,250	3	3	Mg; K
							Pem				
Butts et al. (2007)	II	131	130	ECOG: 0–2	NA	NSCLC	Cis/Car+Gem+Cet;	400,250	3	4	Mg
							Cis/Car+Gem				
Govindan et al. (2011)	II	109	103	ECOG: 0–1	32	NSCLC	Car+Pem+RT+Cet;	400,250	3	2	K
							Car+Pem+RT				
Pirker et al. (2009)	III	1861	1110	ECOG: 0–2	23.8	NSCLC	Cis+Vin+Cet;	400,250	2	5	K
							Cis+Vin				
Lynch et al. (2010)	III	676	645	ECOG: 0–1	NA	NSCLC	Pac/Doc+Car+Cet;	400,250	3	2	Mg
							Pac/Doc+Car				
Burtness et al. (2005)	III	123	116	ECOG: 0–1	31	Head and neck cancer	Cis+Cet;	200,125	2	5	Mg; K; Na
							Cis+Placebo				
Vermorken et al. (2008)	III	477	434	Karnofsky: 70–100	19.1	Head and neck cancer	Cis/Car+FU+Cet;	400,250	NA	3	Mg; K; Ca
							Cis/Car+FU				
Vermorken et al. (2013)	III	765	650	ECOG: 0–1	44 weeks	Head and neck cancer	Cis+FU+Pan;	9	3	3	Mg; K; Ca
					35 weeks		Cis+FU				
Crosby et al. (2013)	II/III	540	258	WHO: 0–1	16.8	Esophageal cancer	Cis+Cap+RT+Cet;	400,250	3	3	Mg; K; Na
							Cis+Cap+RT				
Waddell et al. (2013)	III	575	542	WHO: 0–2	5.3	Esophageal cancer	Epi+Oxa+Cap+Pan;	9	3	3	Mg; K
					4.6						
							Epi+Oxa+Cap				
Okines et al. (2010)	II/III	38	29	WHO: 0–2	NA	Esophageal cancer	Epi+Oxa+Cap+Pan;	9	3	2	K
							Epi+Oxa+Cap				
Philip et al. (2010)	III	766	716	Zubrod: 0–2	NA	Pancreatic cancer	Gem+Cet;	400,250	3	3	K
							Gem				
Lordick et al. (2013)	III	1191	882	ECOG: 0–1	22.4	Gastric cancer	Cap+Cisp+Cet;	400,250	3	3	Mg; K; Ca; Na
					21		Cap+Cis				
Baselga et al. (2013)	II	201	171	ECOG: 0–2	NA	Breast cancer	Cis+Cet;	400,250	3	3	Mg
							Cet				

*NA* not available, *NSCLC* non-small-cell lung cancer, *CTCAE* National Cancer Institute Common Terminology Criteria, *AE* adverse event, *Mg* hypomagnesemia, K hypokalemia, *Ca* hypocalcemia, *Na* hyponatremia, *ECOG* Eastern Cooperative Oncology Group performance status, *WHO* World Health Organization performance status, *BSC* best support care, *Cet* cetuximab, *Cap* capecitabine, *Oxa* oxaliplatin, *Bev* bevacizumab, *FU* fluorouracil, *Leu* leucovorin, *Len* lenalidomide, *Iri* irinotecan, *Pan* panitumumab, *Pem* pemetrexed, *Cis* cisplatin, *Car* carboplatin, *Gem* gemcitabine, *RT* radiotherapy, *Vin* vinorelbine, *Doc* docetaxel, *Epi* epirubicin

^a^The number enrolled is the number of patients recruited for the original study the number analyzed is the number of patients actually exposed to the study

^b^Cetuximab dosage is 400 mg/m^2^ at first dose and 250 mg/m^2^ weekly or 500 mg/m^2^ every 2 weeks; panitumumab dosage is 6 or 9 mg/kg on day 1 every 2 weeks

^c^Study quality was assessed according to the Jadad scale as described in the “Methods” section

### Incidence of electrolyte disorder events

#### Incidence of hypomagnesemia events

**Fig. 2:**
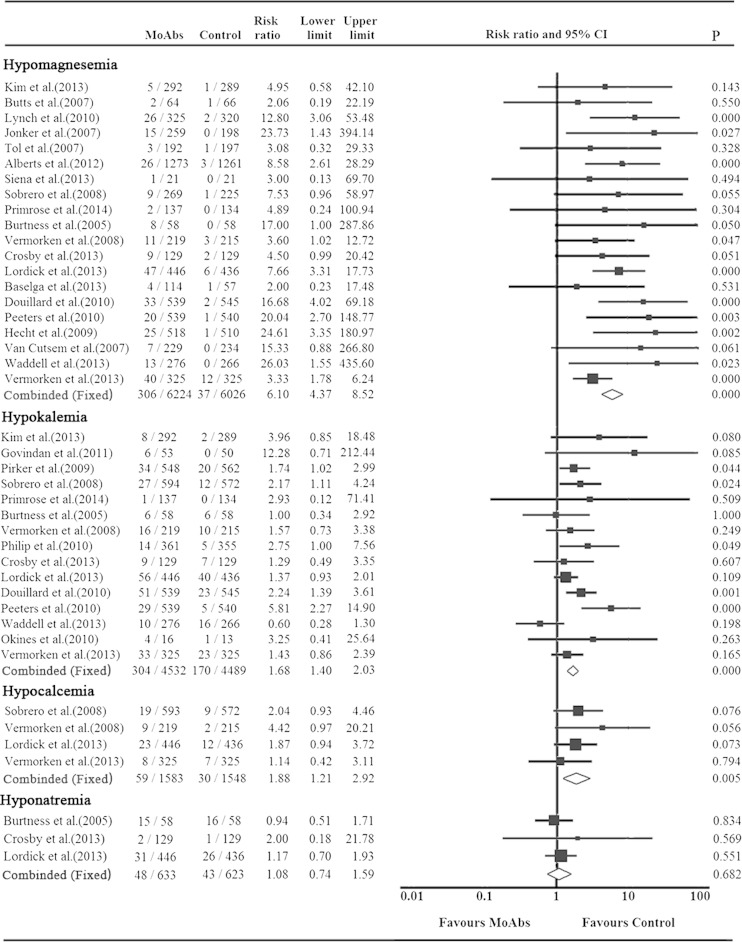
The overall relative risk of different grade 3/4 electrolyte disorder events associated with MoAbs

Twenty RCTs reported grade 3/4, and ten reported all-grade hypomagnesemia events. All-grade hypomagnesemia events were recorded in 879 of 2682 patients in MoAbs-treated group, conferring an incidence of 34.0 % (95 % CI 28.0–40.5 %), whereas that in controls was 9.7 % (95 % CI 6.5–14.3 %) (Table 2), indicating a higher risk of all-grade hypomagnesemia events related to MoAbs (RR 3.37, 95 % CI 2.41–4.72, *p* < 0.001) (Online Resource Fig. 1). The incidence of grade 3/4 hypomagnesemia events in MoAbs was also significantly higher than control group (incidence 4.8 %, 95 % CI 3.6–6.4 %, vs 0.7 %, 95 % CI 0.4–1.2 %, *p* < 0.001), with RR value of 6.10 (95 % CI 4.37–8.52, *p* < 0.001) (Fig. 2).

**Table 2:** Incidence of grade 3/4 or all-grade hypomagnesemia events with MoAbs according to tumor types and MoAbs agents

	Groups	No.	No. of grade 3/4 events/total no.		Incidence (95 % CI)^a^		*p* value
			MoAbs	Control	MoAbs	Control	
Grade 3/4							
Cetuximab	Overall	14	168/3798	21/3606	4.4 (2.9–6.7)	0.8 (0.6–1.3)	<0.001
	Colorectal cancer	6	56/2151	5/2036	2.9 (1.7–4.7)	0.4 (0.2–0.8)	<0.001
	NSCLC	3	33/681	4/675	3.7 (1.2–11.1)	0.7 (0.3–1.8)	<0.001
	Head and neck cancer	2	19/277	3/273	8.3 (3.0–21.0)	1.3 (0.5–3.6)	0.001
	Esophageal cancer	1	9/129	2/129	7.0 (3.7–12.9)	1.6 (0.4–6.0)	0.031
	Gastric cancer	1	47/446	6/436	10.5 (8.0–13.7)	1.4 (0.6–3.0)	<0.001
	Breast cancer	1	4/114	1/57	3.5 (1.3–9.0)	1.8 (0.2–11.4)	0.521
Panitumumab	Overall	6	138/2426	16/2420	5.4 (3.5–8.3)	0.4 (0.1–1.9)	<0.001
	Colorectal cancer	4	85/1825	4/1829	4.6 (3.5–6.1)	0.3 (0.1–0.6)	<0.001
	Esophageal cancer	1	13/276	0/266	4.7 (2.8–7.9)	0.2 (0.0–2.9)	<0.001
	Head and neck cancer	1	40/325	12/325	12.3 (9.2–16.3)	3.7 (2.1–6.4)	<0.001
Overall		20	306/6224	37/6026	4.8 (3.6–6.4)	0.7 (0.4–1.2)	<0.001
All-grade							
Cetuximab	Overall	7	559/1659	188/1545	34.9 (25.9–45.1)	12.6 (9.0–17.3)	<0.001
	Colorectal cancer	4	329/857	91/754	35.8 (24.3–49.1)	12.0 (9.0–16.0)	<0.001
	NSCLC	2	97/356	36/355	37.0 (10.3–75.1)	13.7 (2.5–49.7)	<0.001
	Gastric cancer	1	133/446	61/436	29.8 (25.8–34.2)	14.0 (11.0–17.6)	<0.001
Panitumumab	Overall	3	320/1023	57/1010	31.8 (27.9–36.0)	3.8 (0.7–17.0)	<0.001
	Colorectal cancer	2	231/747	16/744	31.9 (25.4–39.1)	1.8 (0.6–5.4)	<0.001
	Esophageal cancer	1	89/276	41/266	32.2 (27.0–38.0)	15.4 (11.6–20.3)	<0.001
Overall		10	879/2682	245/2555	34.0 (28.0–40.5)	9.7 (6.5–14.3)	<0.001

*MoAbs* monoclonal antibodies, *CI* confidence interval, *NSCLC* non-small-cell lung cancer

^a^Calculated using the random-effect model (Comprehensive Meta Analysis 2, Biostat)

Incidence of hypomagnesemia events was then calculated for cetuximab and panitumumab trials separately (Table 2). Of note, among cetuximab trials, incidences of all-grade and grade 3/4 hypomagnesemia events in cetuximab group were approximately three times (incidence 34.9 %, 95 % CI 25.9–45.1 %, vs 12.6 %, 95 % CI 9.0–17.3 %) and 5.5 times (incidence 4.4 %, 95 % CI 2.9–6.7 %, vs 0.8 %, 95 % CI 0.6–1.3 %) higher than in controls (*p* < 0.001 for both) (Table 2). In panitumumab trials, the effects of hypomagnesemia events were also obvious, all-grade incidence of panitumumab group and control group: 31.8 %, 95 % CI 27.9–36.0 %, vs 3.8 %, 95 % CI 0.7–17.0 %; grade 3/4 incidence 5.4 %, 95 % CI 3.5–8.3 %, vs 0.4 %, 95 % CI 0.1–1.9 % (Table 2). Then, the trials included were stratified for underlying malignant disease. The incidence of grade 3/4 hypomagnesemia events related to cetuximab in colorectal cancer trials was 2.9 % (95 % CI 1.7–4.7 %) [14–20]. While the incidence in panitumumab group was higher with 4.6 % (95 % CI 3.5–6.1 %) (Table 2) [21–24].

#### Incidence of hypokalemia events

Sixteen RCTs reported grade 3/4 and six RCTs reported all-grade hypokalemia events. The grade 3/4 events of anti-EGFR MoAbs were noted in 304 of 4543 patients, yielding the incidence of 6.7 % (95 % CI 5.2–8.7 %), whereas in the control group, the incidence was 3.7 % (95 % CI 2.5–5.4 %) (Online Resource Table 2), implying that addition of anti-EGFR MoAbs increased the risk of hypokalemia (RR = 1.68, 95 % CI 1.40–2.03; *p* < 0.001) (Fig. 2). Similarly, incidence of all-grade hypokalemia in MoAbs group (14.5 %, 95 % CI 8.2–24.4 %) was higher than controls (9.7 %, 95 % CI 6.0–15.2 %, *p* < 0.001) (Online Resource Table 2).

All-grade hypokalemia events with cetuximab occurred in 12.6 % patients and grade 3/4 incidence was 6.1 %, both of which were significantly higher than their controls (*p* < 0.001 for both) (Online Resource Table 2). In subanalysis of different tumor types, the addition of cetuximab augmented notably the incidence of grade 3/4 events in colorectal cancer (2.7, 95 % CI 0.8–8.3) and NSCLC (5.7, 95 % CI 2.9–10.9). For panitumumab treatment group, colorectal cancer patients received an obviously higher incidence of grade 3/4 events than those in controls (7.2 vs 2.1 %, *p* < 0.001).

#### Incidence of hypocalcemia or hyponatremia events

Four RCTs recorded grade 3/4 and two RCTs recorded all-grade hypocalcemia events. The incidence of all-grade hypocalcemia related to cetuximab was 16.8 % (95 % CI 14.2–19.7 %), while the control was 9.9 % (95 % CI 8.0–12.2 %). And, grade 3/4 hypocalcemia of cetuximab and panitumumab was 3.8 % compared with 2.0 % (Online Resource Table 3).

Three RCTs noted grade 3/4, and one noted all-grade hyponatremia events associated with anti-EGFR MoAbs treatment (all of them were in cetuximab trials). The overall incidence of grade 3/4 and all-grade events was 7.8 % (95 % CI 2.1–25.0 %) and 9.4 % (95 % CI 7.0–12.5 %), respectively. However, no significant difference was found (*p* > 0.05 for both) (Online Resource Table 4).

### Relative risk of grade 3/4 electrolyte disorder events

#### Relative risk of grade 3/4 hypomagnesemia events

**Fig. 3:**
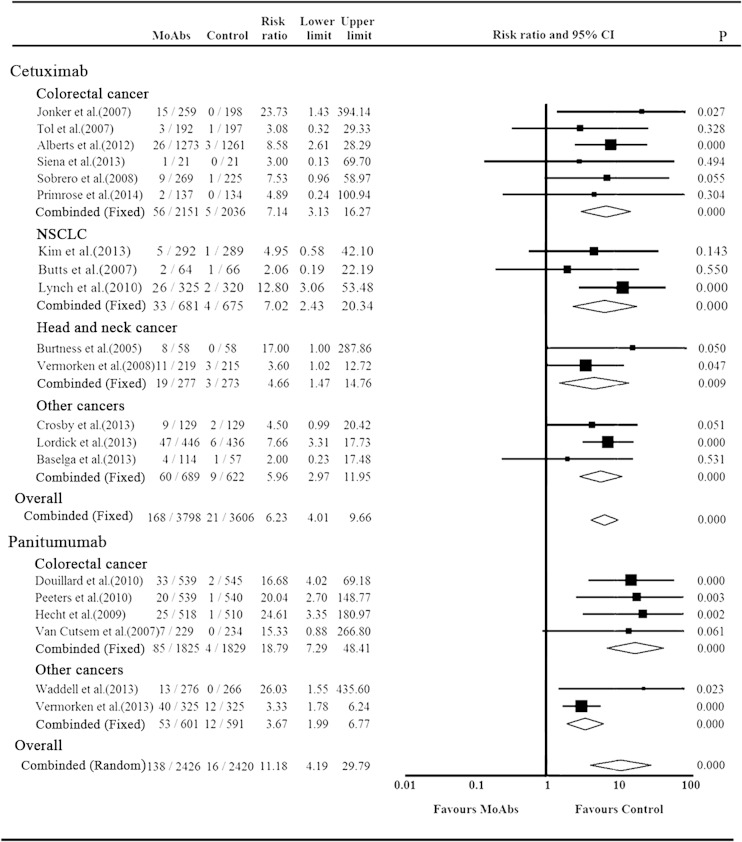
Relative risk of grade 3/4 hypomagnesemia events stratified by tumor types and MoAbs agents

As an exploratory analysis, patients were stratified according to anti-EGFR MoAbs (cetuximab or panitumumab) and underlying malignant disease. Studies of cetuximab showed that 14 RCTs were available to calculate the RR of grade 3/4 hypomagnesemia events. Events treated with or without cetuximab were 168/3798 and 21/3606 (RR 6.23, 95 % CI 4.01–9.66, *p* < 0.001) and the *p* value of Cochrane’s *Q* statistic was 0.93 (*I* ^2^ = 0), justifying the use of the fixed effect model (Mantel–Haenszel) (Fig. 3). Six RCTs of panitumumab contained 138 grade 3/4 hypomagnesemia events in 2426 patients compared with 16 events in 2420 patients in control (RR 11.18, 95 % CI 4.20–29.80, *p* < 0.001) (Fig. 3) using random effect model. The heterogeneity existed (*p* = 0.072, *I* ^2^ = 50.7 %), and sensitivity analysis was conducted. It seemed that when we omitted the study conducted by Vermorken et al., the heterogeneity decreased to 0 % (*p* = 0.997), though the result was precarious with RR of 19.42 (95 % CI 7.92–47.63, *p* < 0.001) (Fig. 3).

When stratifying for underlying cancers, we noted the RR of grade 3/4 hypomagnesemia events varied either in cetuximab trials (*p* < 0.001) or panitumumab trials (*p* = 0.004, data not shown). Subanalysis showed that colorectal cancer patients had the highest RR: 7.14 of cetuximab (95 % CI 3.13–16.27, *p* < 0.001) and 18.79 of panitumumab (95 % CI 7.29–48.41, *p* < 0.001) (Fig. 3). Significant statistical differences were also observed in NSCLC (RR = 7.02, 95 % CI 2.43–20.34, *p* < 0.001), head and neck cancer (4.66, 95 % CI 1.47–14.76, *p* = 0.009) in cetuximab trials, as well as non-colorectal cancer in panitumumab trials (3.67, 95 % CI 1.99–6.77, *p* < 0.001) (Fig. 3).

#### Relative risk of grade 3/4 hypokalemia events

**Fig. 4:**
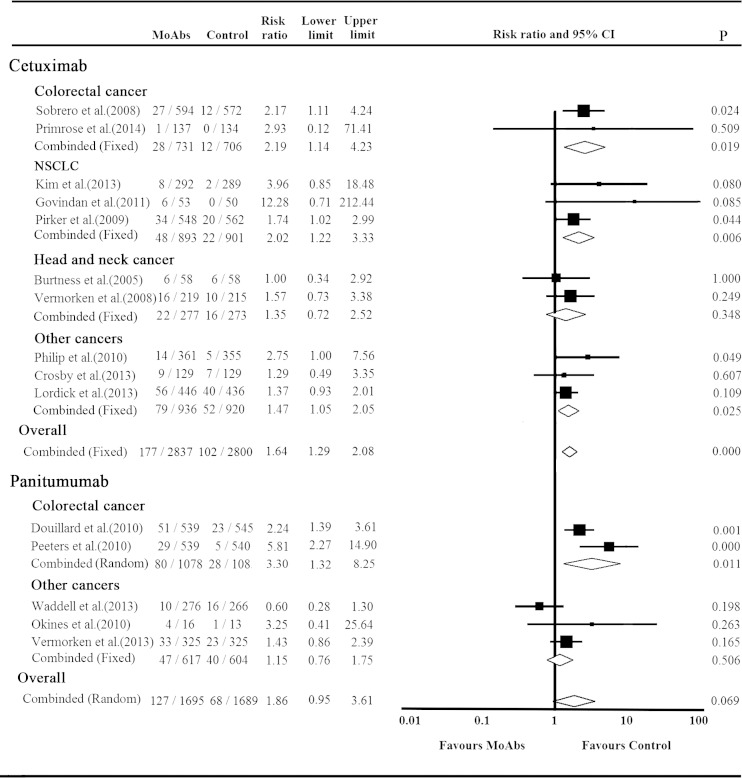
Relative risk of grade 3/4 hypokalemia events stratified by tumor types and MoAbs agents

Upon stratification by MoAbs agents, we observed that the RR of grade 3/4 hypokalemia events was 1.64 (95 % CI 1.29–2.08, *p* < 0.001) for cetuximab-based regimens and 1.86 (95 % CI 0.95–3.61, *p* = 0.069) for panitumumab-based regimens (Fig. 4). No heterogeneity was detected among ten cetuximab trials (*p* = 0.642, *I* ^2^ = 0). As some trials had a wide variation in confidence intervals in panitumumab subgroup, which could decline the precision of pooled results, thus a sensitivity analysis was conducted to examine the stability and reliability of the overall RRs by sequential omission of individual studies. When the study results reported by Waddell et al. was omitted [34], which seemed to explain the heterogeneity among those studies, the RR value turned into 2.40 (95 % CI 1.36–4.24) and the heterogeneity among the remaining studies decrease to 56 % (*p* = 0.076).

RRs of grade 3/4 hypokalemia in cetuximab trials were significantly different between tumor types (*p* < 0.001) but were similar in panitumumab trials (*p* = 0.239, data not shown). Colorectal cancer patients presented the lower level of RR in cetuximab-based therapy (RR = 2.19, 95 % CI 1.14–4.23, *p* = 0.019) than in panitumumab-based therapy (RR = 3.30, 95 % CI 1.32–8.25, *p* = 0.011) (Fig. 4). And, significant statistical difference was also observed in NSCLC patients in cetuximab trials (*p* = 0.006). All the subgroup analysis were judged to use fixed effect model due to the *p* values of Cochrane’s *Q* statistic of >0.1, except those colorectal cancer patients treated with panitumumab with the *p* value of 0.077 (*I* ^2^ = 68.0 %), which was calculated using random effect model.

#### Relative risk of grade 3/4 hypocalcemia or hyponatremia events

Three RCTs reported grade 3/4 hypocalcemia related to cetuximab, and only one RCT recorded the events with panitumumab. Patients with cetuximab-based therapy had a significantly higher risk of electrolyte disorders (RR = 2.12, 95 % CI 1.30–3.45, *p* = 0.003) (Online Resource Fig. 2), whereas panitumumab did not increase this events as reported (RR = 1.14, 95 % CI 0.42–3.12, *p* = 0.794) (Online Resource Fig. 2).

The analysis of grade 3/4 hyponatremia events showed that RR in cetuximab trials was 1.08 (95 % CI 0.74–1.59, *p* = 0.682) (Online Resource Fig. 3), but no one reported the events of panitumumab. Due to a lack of sufficient studies, subgroup analysis of different tumor types was not conducted.

### Publication bias

The publication bias was performed in the pooling analysis of risk of grade 3/4 hypomagnesemia events and hypokalemia events associated with cetuximab due to the fact that the included studies were more than ten. Evidence of publication bias was not detected by either Begg’s test or Egger’s test (RR of hypomagnesemia event: Begg’s test *p* = 0.584, Egger’s test *p* = 0.441, RR of hypokalemia event: Begg’s test *p* = 0.152, Egger’s test *p* = 0.074, respectively)

## Discussion

Studies that investigated the toxicity of anti-EGFR MoAbs mainly focused on the common adverse events, such as skin rash [39], hematologic toxicity [40], and specific electrolyte disturbance like hypomagnesemia [41]. To our best knowledge, seldom study has synthetically studied the incidence and risk of all-grade and grade 3/4 electrolyte disorders of cetuximab- or panitumumab-related therapy. As EGFR can be overexpressed in a wide range of tumors and it correlates with poor survival and cancer progression, inhibition of EGFR signaling pathway will be a promising therapeutic target [42]. However, cetuximab and panitumumab both bind with high affinity to human EGFR and hence could reinforce the cytotoxic effects of conventional chemotherapy or chemoradiotherapy [43]. Therefore, the overall benefits of anti-EGFR MoAbs remain to be confirmed. Electrolyte disorders are quite common in overwhelming majority of cancer patients and may result in serious adverse events. Adequate recognition and management of electrolyte disorders is important for those patients who receive anti-EGFR MoAbs therapy. However, the relationship between grade 3/4 electrolyte disorder events and MoAbs-based therapy is difficult to evaluate in individual RCTs for a lack of enough patients.

In our study, data from 25 RCTs was pooled to overcome this limitation and the results demonstrated that therapy with anti-EGFR MoAbs can dramatically increased the risk of grade 3/4 hypomagnesemia events (RR = 6.10, 95 % CI 4.37–8,52, *p* < 0.001; incidence compared with controls: 4.8 vs 0.7 %). And, all-grade events reached as high as 34.0 % in MoAbs-treated group, compared with 9.7 % in controls (*p* < 0.001). Colorectal cancer patients had the highest risk of grade 3/4 hypomagnesemia events among cancer patients (cetuximab: RR = 7.14; panitumumab: RR = 18.79). Meanwhile, MoAbs also obviously increased RR of grade 3/4 hypokalemia and hypocalcemia events with the value of 1.68 and 1.88, respectively. Interestingly, colorectal cancer patients were also more prone to have grade 3/4 hypokalemia events than others (cetuximab: RR = 2.19; panitumumab: RR = 3.30), which was similar with that of grade 3/4 hypomagnesemia events. However, no obviously higher risk of hyponatremia events related to MoAbs was discovered in our study. In brief, the risk of electrolyte disorder events was dramatically increased if the anti-EGFR therapy was added. Therefore, more attention should be paid to the electrolyte disorders when patients treated with anti-EGFR MoAb agents alone or combined with chemotherapy, whereas the mechanism behind these toxicities has not yet been well identified.

Recent studies tend to suggest that a new Mg^2+^ permeable channel TRPM6 (transient receptor potential cation channel, subfamily M, member 6) and TRPM7 were involved in transepithelial Mg^2+^ transport in the distal convoluted tubule [44]. And, pro-EGF and TRPM6 were both predominantly expressed in distal convoluted tubule, which was the main site of active renal Mg^2+^ reabsorption. However, the stimulatory effect of EGF on TRPM6 activity could be diminished by preincubation of cetuximab, an EGFR blockade, thus affecting Mg^2+^ transport and leading to hypomagnesemia [45]. Hypokalemia may relate to hypomagnesemia through TRPM6 [46]. Increasing potassium is required to repair Na-K-ATPase due to the magnesium deficiency. Then, over-intracellular transport of potassium could result in hypokalemia. Thus, on the contrary, sodium is mainly unaltered due to the extracellular transport. Human TRPM6 also give rise to hypomagnesemia with secondary hypocalcemia (HSH) [47]. Although the mechanism responsible for development of hypocalcemia is unclear, several explanations such as end-organ unresponsiveness to parathyroid hormone (PTH), altered release of PTH, and impaired formation of 1,25-dihydroxy vitamin D3 are offered. The exact pathogenesis of anti-EGFR MoAbs associated electrolyte disorders remains to be elucidated.

In particular, the grade 3/4 electrolyte disorders risk is higher in colorectal cancer patients yet not the incidence, when compared with other tumors. The differences in the results are still unknown. However, it has to be noted that most trials of other tumors had implemented a treatment with cisplatin as chemotherapy, a well-known harmful agent to renal convoluted tubules. And, it could aggravate the renal function and result in an extensive electrolyte disorders. On the other hand, 70–75 % colorectal cancer patients overexpress EGFR [48], more than other cancer patients like NSCLC with 60 % [49]. Thus, this anti-EGFR treatment might obviously raise the risks of electrolyte disorders with MoAbs as explained previously.

To note that patients received panitumumab therapy were more inclined to have severe electrolyte disorders than cetuximab. The possible reason can be the absolutely high affinity to human EGFR of panitumumab that reinforced its adverse events [43]. Moreover, the longer half-life of panitumumab than cetuximab (7.5 vs 4.7 days) rises the possibility that the different pharmacokinetics of the two MoAbs somehow matters.

For the high risk of severe hypomagnesemia events, most studies mainly focus on the magnesium level in serum. The RR of grade 3/4 hypomagnesemia events associated with cetuximab reported by Petrelli et al. was 9.81 [41], higher than 8.6 from Chen et al. [50] and 6.23 from our study. And, the RR related with panitumumab in Petreli’s study was 11.68, which was similar to the result of our result. The controversial differences may be rooted in different qualities of clinical trials included and some updated trials that were not included in the previous studies.

However, despite the size of this meta-analysis, several limitations are still worth considering. First, these studies were conducted at major academic institutions among patients in hospital and with adequate major organ function, which may neglect outpatients and patients with organ dysfunction, thus could not reflect the general patient population. Second, the process of recording the electrolyte disorder events varies in each clinical trial since these events were not the primary end point of their trials. Therefore, a bias of reported incidence rates emerges. Publication bias of risk of hypomagnesemia and hypokalemia analysis in cetuximab setting was not detected according to Begg’s and Egger’s test. However, it should be interpreted with caution, as these methodologies are not fully bias-free. Since the publication bias test was only performed on the cetuximab setting which included more than ten studies, there might be some evidences of publication bias in the remaining analyses. Third, this is a meta-analysis at study level, so we can not solve the confounding factors that inherent in those included trials due to the inadequate control and thus could result in potential bias and toward exaggeration or underestimation of risk estimates, such as different chemotherapy exposures, different CTCAE versions and failure to follow-up cases, all of which may lead to the existence of heterogeneity in the incidence of adverse events. Finally, the number of trials that recorded hypocalcemia and hyponatremia events was limited. It is likely that significant differences in hypocalcemia events between treatment arm and controls in panitumumab trials would arise if more studies were available. Nonetheless, all efforts have been made to contain all related trials and all of them are well-conducted. Majority of them are multicenter randomized phase III trials (many are registered studies). This meta-analysis thus pooled a limited but robust “core” of clinical data to draw a final unequivocal result.

## Conclusion

Our results indicated that incidences of electrolyte disorders were obviously elevated with anti-EGFR MoAbs therapy, especially in colorectal cancer patients. Addition of anti-EGFR MoAbs would dramatically increase risk of hypomagnesemia events, as well as hypokalemia and hypocalcemia. Panitumumab seemed to have a higher risk in causing severe electrolyte disorders than cetuximab. Among different cancers, colorectal cancer patients receiving anti-EGFR MoAbs treatment showed the highest risk of electrolyte disorders compared with their controls. However, majority of patients with electrolyte disorders are asymptomatic, although symptoms such as fatigue, muscular cramps, and cardiac arrhythmias could be associated with electrolyte disorders. Given its high incidence and risk, rigorous monitoring and early treatment of electrolyte disorders are proposed.

## Electronic supplementary material

Below is the link to the electronic supplementary material.

Fig 1 (Online Resource) The overall relative risk of different all-grade electrolyte disorder events associated with MoAbs. (GIF 15 kb)

High resolution image (TIFF 6817 kb)

Fig 2 (Online Resource) Relative risk of grade 3/4 hypocalcemia events stratified by MoAbs agents. (GIF 7 kb)

High resolution image (TIFF 4784 kb)

Fig 3 (Online Resource) Relative risk of grade 3/4 hyponatremia events associated with cetuximab. (GIF 7 kb)

High resolution image (TIFF 4741 kb)

Table S1 Definitions of hypomagnesemia, hypokalemia, hypocalcemia and hyponatremia in different CTCAE versions (DOC 46 kb)

Table S2 Incidence of grade 3/4 (A) or all-grade (B) hypokalemia events with MoAbs according to tumor types and MoAbs agents (DOC 54 kb)

Table S3 Incidence of grade 3/4 (A) or all-grade (B) hypocalcemia events with MoAbs according to tumor types and MoAbs agents (DOC 43 kb)

Table S4 Incidence of grade 3/4 (A) or all-grade (B) hyponatremia events with MoAbs according to tumor types and MoAbs agents (DOC 39 kb)
