# Supplementary figures and images for: Electrolyte disorders assessment in solid tumor patients treated with anti-EGFR monoclonal antibodies: a pooled analysis of 25 randomized clinical trials
Source: Tumour Biol. 2014 Dec 28;36(5):3471–82. doi: 10.1007/s13277-014-2983-9 (PMC4445483; doi:10.1007/s13277-014-2983-9)

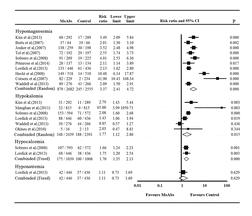

Supplement: Supplementary file 1 — (Online Resource) The overall relative risk of different all-grade electrolyte disorder events associated with MoAbs. (GIF 15 kb) [file 13277_2014_2983_Fig5_ESM.gif]

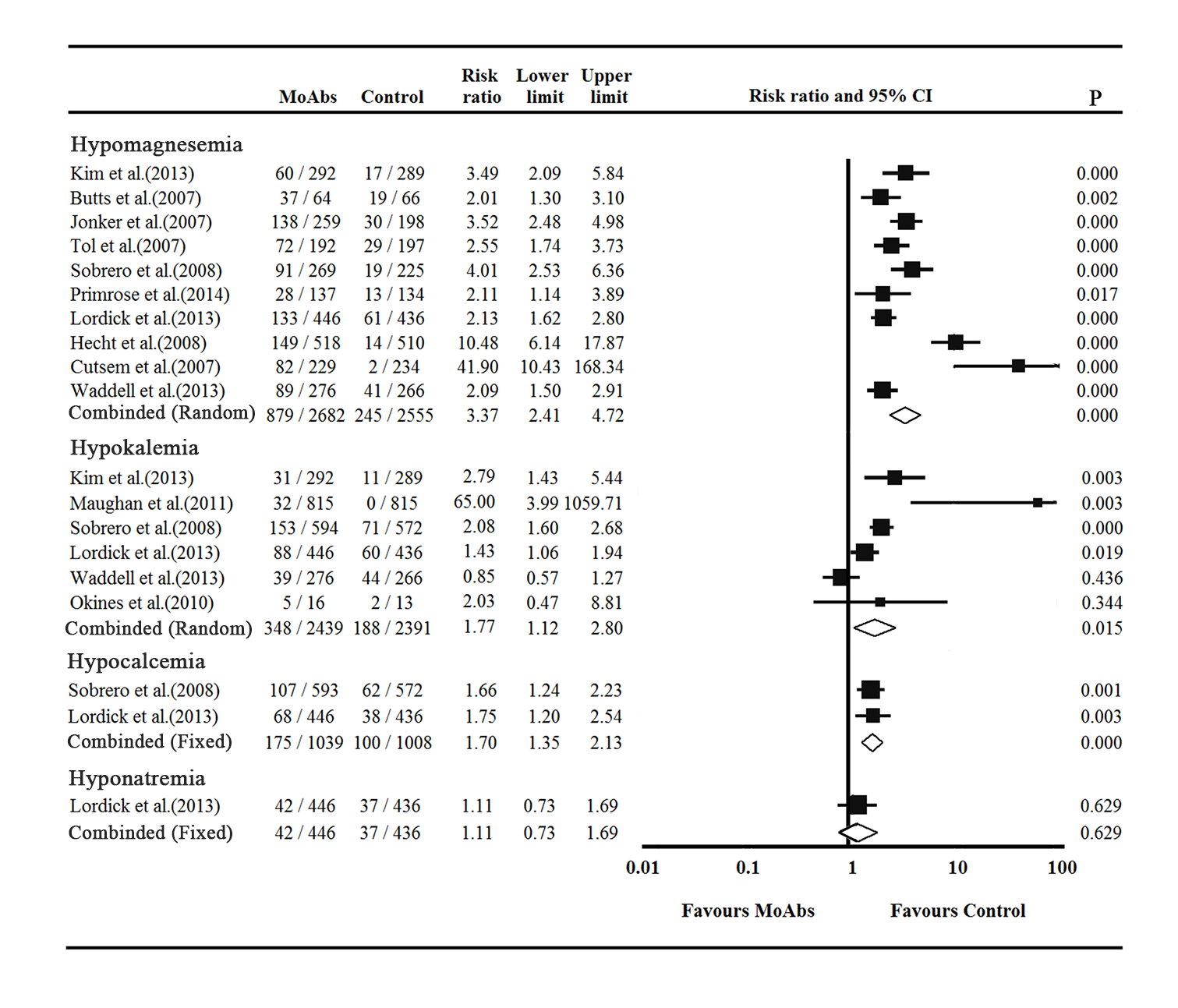

Supplement: Supplementary file 2 — High resolution image (TIFF 6817 kb) [file 13277_2014_2983_MOESM1_ESM.tif]

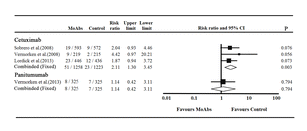

Supplement: Supplementary file 3 — (Online Resource) Relative risk of grade 3/4 hypocalcemia events stratified by MoAbs agents. (GIF 7 kb) [file 13277_2014_2983_Fig6_ESM.gif]

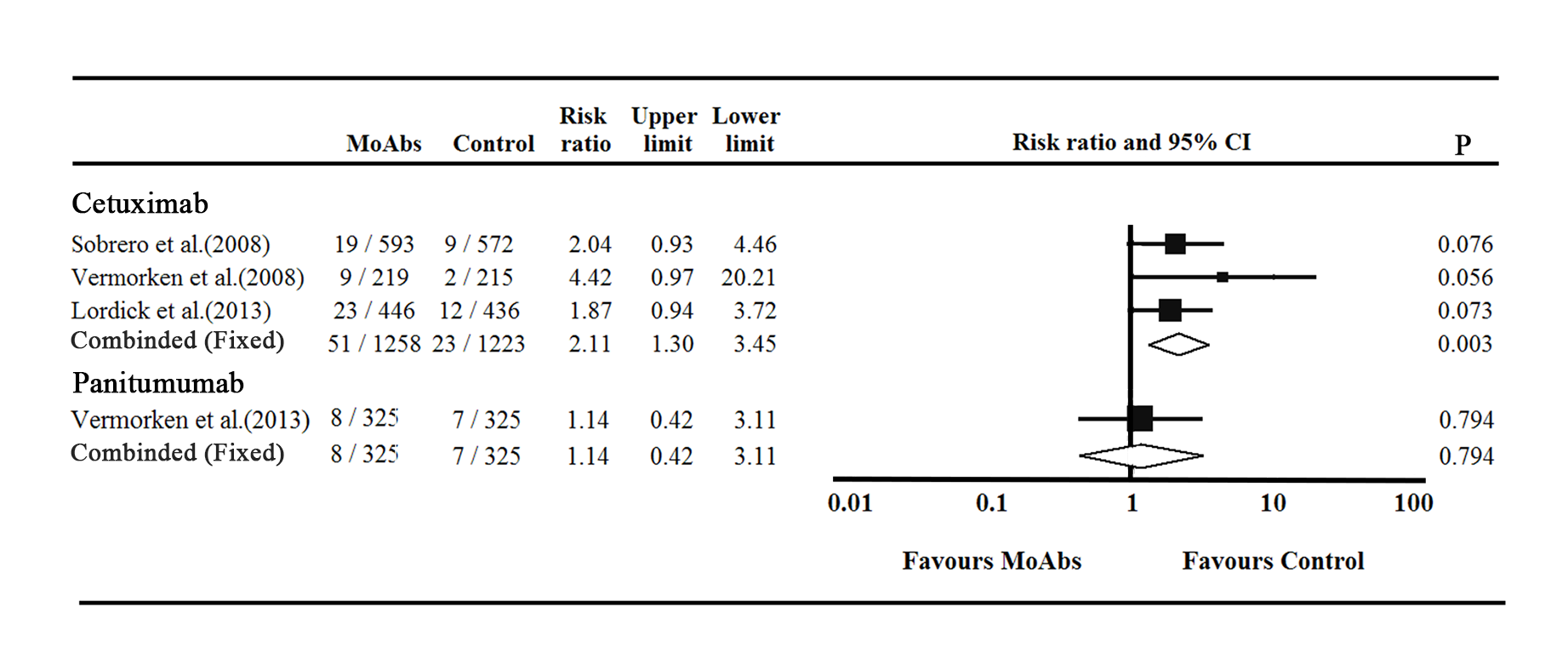

Supplement: Supplementary file 4 — High resolution image (TIFF 4784 kb) [file 13277_2014_2983_MOESM2_ESM.tif]

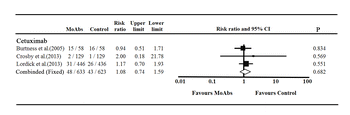

Supplement: Supplementary file 5 — (Online Resource) Relative risk of grade 3/4 hyponatremia events associated with cetuximab. (GIF 7 kb) [file 13277_2014_2983_Fig7_ESM.gif]

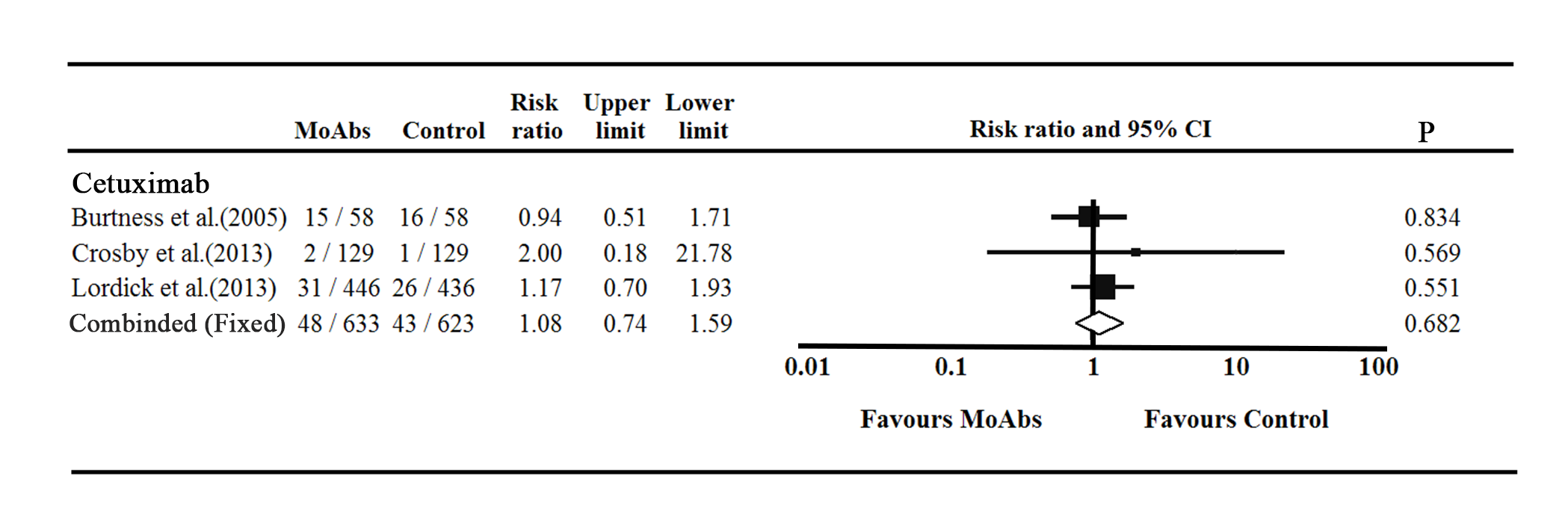

Supplement: Supplementary file 6 — High resolution image (TIFF 4741 kb) [file 13277_2014_2983_MOESM3_ESM.tif]
