# Supplementary material for: Electrolyte disorders assessment in solid tumor patients treated with anti-EGFR monoclonal antibodies: a pooled analysis of 25 randomized clinical trials
Source: Tumour Biol. 2014 Dec 28;36(5):3471–82. doi: 10.1007/s13277-014-2983-9 (PMC4445483; doi:10.1007/s13277-014-2983-9)
Supplement: Supplementary file 7 — Definitions of hypomagnesemia, hypokalemia, hypocalcemia and hyponatremia in different CTCAE versions (DOC 46 kb) [file 13277_2014_2983_MOESM4_ESM.doc]

Table S1 Definitions of hypomagnesemia, hypokalemia, hypocalcemia and hyponatremia in different CTCAE versions

| **CTCAE Version 2** | | | | |
| --- | --- | --- | --- | --- |
|  | Hypomagnesemia (mmol/L) | Hypokalemia (mmol/L) | Hypocalcemia (mmol/L) | Hyponatremia (mmol/L) |
| Grade 1 | <LLN- 0.5 | <LLN- 3.0 | <LLN- 2.0 | <LLN- 130 |
| Grade 2 | <0.5-0.4 | - | <2.0-1.75 | - |
| Grade 3 | <0.4- 0.3 | <3.0-2.5 | <1.75- 1.5 | <130- 120 |
| Grade 4 | < 0.3 | <2.5 | <1.5 | <120 |
| Grade 5 | Death | Death | Death | Death |

*LLN: local laboratory value*

|  | **CTCAE Version 3** | | | |
| --- | --- | --- | --- | --- |
|  | Hypomagnesemia (mmol/L) | Hypokalemia (mmol/L) | Hypocalcemia (mmol/L) | Hyponatremia (mmol/L) |
| Grade 1 | <LLN – 0.5 | <LLN – 3.0 | <LLN – 2; Ionized calcium <LLN – 1.0 | <LLN – 130 |
| Grade 2 | <0.5 – 0.4 | - | <2.0 – 1.75; Ionized calcium <1.0 – 0.9 | - |
| Grade 3 | <0.4 – 0.3 | <3.0 – 2.5 | <1.75 – 1.5; Ionized calcium <0.9 – 0.8 | <130 – 120 |
| Grade 4 | <0.3 | <2.5 | <1.5; Ionized calcium <0.8 | <120 |
| Grade 5 | Death | Death | Death | Death |

*LLN: local laboratory value*

|  | | **CTCAE Version 4** | | | | |
| --- | --- | --- | --- | --- | --- | --- |
|  | Hypomagnesemia (mmol/L) | | Hypokalemia (mmol/L) | Hypocalcemia (mmol/L) | Hyponatremia (mmol/L) |  |
| Grade 1 | <LLN-0.5 | | <LLN-3.0 | <LLN-2.0; Ionized calcium <LLN - 1.0 | <LLN-130 |  |
| Grade 2 | <0.5 - 0.4 | | <LLN - 3.0; Symptomatic; Intervention indicated | <2.0 - 1.75; Ionized calcium < 1.0-0.9; Symptomatic | - |  |
| Grade 3 | <0.4 - 0.3 | | <3.0 - 2.5; Hospitalization indicated | <1.75 - 1.5; Ionized calcium <0.9-0.8; Hospitalization indicated | <130 - 120 |  |
| Grade 4 | <0.3； Life-threatening | | <2.5; Life-threatening | <1.5; Ionized calcium <0.8; Life-threatening | <120; Life-threatening |  |
| Grade 5 | Death | | Death | Death | Death |  |

*LLN: local laboratory value*
