# Supplementary material for: Electrolyte disorders assessment in solid tumor patients treated with anti-EGFR monoclonal antibodies: a pooled analysis of 25 randomized clinical trials
Source: Tumour Biol. 2014 Dec 28;36(5):3471–82. doi: 10.1007/s13277-014-2983-9 (PMC4445483; doi:10.1007/s13277-014-2983-9)
Supplement: Supplementary file 8 — Incidence of grade 3/4 (A) or all-grade (B) hypokalemia events with MoAbs according to tumor types and MoAbs agents (DOC 54 kb) [file 13277_2014_2983_MOESM5_ESM.doc]

Table S2. Incidence of grade 3/4 (A) or all-grade(B) hypokalemia events with MoAbs according to tumor types and MoAbs agents

A

|  |  |  | No. of grade3/4 events / total no. | | Incidence (95%CI) ^1^ | |  |
| --- | --- | --- | --- | --- | --- | --- | --- |
|  | Groups | No. | MoAbs | Control | MoAbs | Control | *p* value |
| Cetuximab | Overall | 11 | 177/2858 | 102/2821 | 6.1(4.2-8.7) | 3.3(1.9-5.6) | <0.001 |
|  | colorectal cancer | 3 | 28/752 | 12/727 | 2.7（0.8-8.3） | 2.0（1.1-3.4） | 0.014 |
|  | NSCLC | 3 | 48/893 | 22/901 | 5.7（2.9-10.9） | 1.7（0.5-5.9） | 0.001 |
|  | head and neck cancer | 2 | 22/277 | 16/273 | 8.0（5.3-11.9） | 6.7（3.0-14.2） | 0.336 |
|  | pancreatic cancer | 1 | 14/361 | 5/355 | 3.9（2.3-6.4） | 1.4（0.6-3.3） | 0.040 |
|  | oesophageal cancer | 1 | 9/129 | 7/129 | 7.0（3.7-12.9） | 5.4（2.6-10.9） | 0.606 |
|  | gastric cancer | 1 | 56/446 | 40/436 | 1.26（9.8-16.0） | 9.2（6.8-12.3） | 0.107 |
| Panitumumab | Overall | 5 | 127/1685 | 68/1689 | 8(5.2-12.1) | 4.2(2.3-7.4) | <0.001 |
|  | colorectal cancer | 2 | 80/1078 | 28/1085 | 7.2 (4.1-12.4) | 2.1 (0.5-8.9) | <0.001 |
|  | oesophageal cancer | 2 | 14/292 | 17/279 | 9.6 (1.2-47.3) | 6.1 (3.8-9.6) | 0.494 |
|  | head and neck cancer | 1 | 33/325 | 23/325 | 10.2 (7.3-13.9) | 7.1 (4.7-10.4) | 0.162 |
| Overall |  | 16 | 304/4543 | 170/4510 | 6.7(5.2-8.7) | 3.7(2.5-5.4) | <0.001 |

B

|  |  |  | No. of all-grade events / total no. | | Incidence (95%CI) ^1^ | |  |
| --- | --- | --- | --- | --- | --- | --- | --- |
|  | Groups | No. | MoAbs | Control | MoAbs | Control | *p* value |
| Cetuximab | Overall | 4 | 304/2147 | 142/2112 | 12.6(5.8-25.3) | 7.0(3.5-13.5) | <0.001 |
|  | colorectal cancer | 2 | 185/1409 | 71/1387 | 10.7(1.5-49.3) | 1.1(0.0-69.6) | <0.001 |
|  | NSCLC | 1 | 31/292 | 11/289 | 10.6(7.6-14.7) | 3.8(2.1-6.7) | 0.002 |
|  | gastric cancer | 1 | 88/446 | 60/436 | 19.7(16.3-23.7) | 13.8(10.8-17.3) | 0.018 |
| Panitumumab | Overall | 2 | 44/292 | 46/279 | 19.4(8.4-38.7) | 16.5(12.6-21.3) | 0.642 |
|  | oesophageal cancer | 2 | 44/292 | 46/279 | 19.4(8.4-38.7) | 16.5(12.6-21.3) | 0.642 |
| Overall |  | 6 | 348/2439 | 188/2391 | 14.5(8.2-24.4) | 9.7(6.0-15.2) | <0.001 |

MoAbs, monoclonal antibodies; CI, confidence interval; NSCLC, non-small cell lung cancer; ^1^Calculated using the random-effect model (Comprehensive Meta Analysis 2, Biostat)
