# Supplementary material for: Electrolyte disorders assessment in solid tumor patients treated with anti-EGFR monoclonal antibodies: a pooled analysis of 25 randomized clinical trials
Source: Tumour Biol. 2014 Dec 28;36(5):3471–82. doi: 10.1007/s13277-014-2983-9 (PMC4445483; doi:10.1007/s13277-014-2983-9)
Supplement: Supplementary file 9 — Incidence of grade 3/4 (A) or all-grade (B) hypocalcemia events with MoAbs according to tumor types and MoAbs agents (DOC 43 kb) [file 13277_2014_2983_MOESM6_ESM.doc]

Table S3 Incidence of grade 3/4 (A) or all-grade (B) hypocalcemia events with MoAbs according to tumor types and MoAbs agents

A

|  | Groups | No. | No. of grade 3/4 events / total no. | | Incidence (95%CI) ^1^ | | *p* value |
| --- | --- | --- | --- | --- | --- | --- | --- |
|  |  |  | MoAbs | Control | MoAbs | Control |  |
| Cetuximab | Overall | 3 | 51/1258 | 23/1223 | 4.1(3.0-5.6) | 1.9(1.1-3.2) | 0.001 |
|  | colorectal cancer | 1 | 19/593 | 9/572 | 3.2(2.1-5.0) | 1.6(0.8-3.0 ) | 0.069 |
|  | head and neck cancer | 1 | 9/219 | 2/215 | 4.1(2.2-7.7) | 0.9(0.2-3.6) | 0.035 |
|  | gastric cancer | 1 | 23/446 | 12/436 | 5.2(3.5-7.6) | 2.8(1.6-4.8) | 0.067 |
| Panitumumab | Overall | 1 | 8/325 | 7/325 | 2.5(1.2-4.8) | 2.2(1.0-4.4) | 0.794 |
|  | head and neck cancer | 1 | 8/325 | 7/325 | 2.5(1.2-4.8) | 2.2(1.0-4.4) | 0.794 |
| Overall |  | 4 | 59/1583 | 30/1548 | 3.8(2.8-5.2) | 2.0(1.4-2.9) | 0.003 |

B

|  | Groups | No. | No. of all-grade events / total no. | | Incidence (95%CI)^1^ | | *p* value |
| --- | --- | --- | --- | --- | --- | --- | --- |
|  |  |  | MoAbs | Control | MoAbs | Control |  |
| Cetuximab | Overall | 2 | 175/1039 | 100/1008 | 16.8(14.2-19.7) | 9.9(8.0-12.2) | <0.001 |
|  | colorectal cancer | 1 | 107/593 | 62/572 | 18.0(15.2-21.3) | 10.8(8.5-13.7) | <0.001 |
|  | gastric cancer | 1 | 68/446 | 38/436 | 15.2(12.2-18.9) | 8.7(6.4-11.8) | 0.003 |
| Overall | / | 2 | 175/1039 | 100/1008 | 16.8(14.2-19.7) | 9.9(8.0-12.2) | <0.001 |

MoAbs, monoclonal antibodies; CI, confidence interval; NSCLC, non-small cell lung cancer; ^1^Calculated using the random-effect model (Comprehensive Meta Analysis 2, Biostat)
