# Supplementary material for: Electrolyte disorders assessment in solid tumor patients treated with anti-EGFR monoclonal antibodies: a pooled analysis of 25 randomized clinical trials
Source: Tumour Biol. 2014 Dec 28;36(5):3471–82. doi: 10.1007/s13277-014-2983-9 (PMC4445483; doi:10.1007/s13277-014-2983-9)
Supplement: Supplementary file 10 — Incidence of grade 3/4 (A) or all-grade (B) hyponatremia events with MoAbs according to tumor types and MoAbs agents (DOC 39 kb) [file 13277_2014_2983_MOESM7_ESM.doc]

Table S4 Incidence of grade 3/4 (A) or all-grade (B) hyponatremia events with MoAbs according to tumor types and MoAbs agents

A

|  | Groups | No. | No. of grade 3/4 events / total no. | | Incidence (95%CI) ^1^ | | *p* value |
| --- | --- | --- | --- | --- | --- | --- | --- |
|  |  |  | MoAbs | Control | MoAbs | Control |  |
| Cetuximab | Overall | 3 | 48/633 | 43/623 | 7.8(2.1-25) | 6.9(1.4-27.1) | 0.642 |
|  | head and neck cancer | 1 | 15/58 | 16/58 | 25.9(16.2-38.6) | 27.6(17.6-40.4) | 0.834 |
|  | oesophageal cancer | 1 | 2/129 | 1/129 | 1.6(0.4-6.0) | 0.8(0.1-5.3) | 0.561 |
|  | gastric cancer | 1 | 31/446 | 26/436 | 7.0(4.9-9.7) | 6.0(4.1-8.6) | 0.551 |
| Overall |  | 3 | 48/633 | 43/623 | 7.8(2.1-25.0) | 6.9(1.4-27.1) | 0.642 |

B

|  | Groups | No. | No. of all-grade events / total no. | | Incidence (95%CI)^1^ | | *p* value |
| --- | --- | --- | --- | --- | --- | --- | --- |
|  |  |  | MoAbs | Control | MoAbs | Control |  |
| Cetuximab | Overall | 1 | 42/446 | 37/436 | 9.4(7.0-12.5) | 8.5 (6.2-11.5) | 0.628 |
|  | gastric cancer | 1 | 42/446 | 37/436 | 9.4(7.0-12.5) | 8.5 (6.2-11.5) | 0.628 |
| Overall |  | 1 | 42/446 | 37/436 | 9.4(7.0-12.5) | 8.5 (6.2-11.5) | 0.628 |

MoAbs, monoclonal antibodies; CI, confidence interval; NSCLC, non-small cell lung cancer; ^1^Calculated using the random-effect model (Comprehensive Meta Analysis 2, Biostat)
